# Comparing the Outcomes Between Minimal Incision Endoscopic Cubital Tunnel Release and Open Cubital Tunnel Release: A Prospective Randomized Controlled Trial

**DOI:** 10.7759/cureus.115413

**Published:** 2026-08-29

**Authors:** Nuttapath Tepsen, Jaruwat Vechasilp, Thanapol Wangrattanapranee, Lertkong Nitiwarangkul, Woraphon Jaroenporn

**Affiliations:** 1 Orthopaedics, Police General Hospital, Bangkok, THA

**Keywords:** cubital tunnel syndrome, endoscopic cubital tunnel release, open cubital tunnel release, postoperative pain, scar sensitivity, time return to work

## Abstract

Cubital tunnel syndrome is the second most common peripheral nerve entrapment in the upper extremity requiring surgical intervention. While endoscopic decompression techniques have gained popularity due to presumed faster recovery and reduced morbidity, data comparing long-term outcomes remain limited. This study introduces a novel minimal incision endoscopic cubital tunnel release (MICuR) technique and evaluates its efficacy against conventional open cubital tunnel release (OCuTR).

In this prospective randomized controlled trial, 30 patients with moderate to severe cubital tunnel syndrome were randomized to undergo either MICuR (n=15) or OCuTR (n=15). Postoperative outcomes were assessed at multiple intervals up to six months and included postoperative pain (Visual Analog Scale (VAS)), functional recovery (modified Bishop score), scar sensitivity (Modified Vancouver Scar Scale (mVSS)), time to return to work, and complications.

The results of this study showed that MICuR significantly reduced early postoperative pain compared to OCuTR, with VAS scores of 0.8 vs. 4.7 on postoperative day 1 (p<0.01) and consistently lower pain scores through day 6 (all p<0.01). Time to return to work was markedly shorter in the MICuR group (two days vs. 13 days; p<0.01). Scar sensitivity was also significantly reduced at one, three, and six months (p<0.01). Functional recovery, as measured by the modified Bishop score, was comparable between groups at all timepoints. No significant differences in complication rates were observed.

In conclusion, MICuR offers superior short-term outcomes in terms of pain control, scar sensitivity, and return to work, without compromising functional recovery or increasing complications. These findings support MICuR as a promising alternative for patients seeking rapid recovery and improved cosmetic outcomes.

## Introduction

Cubital tunnel syndrome is the second most common peripheral nerve entrapment in the upper extremity, with an annual incidence of 20.9 per 100,000 individuals [1]. It results from ulnar nerve compression at the cubital tunnel, typically presenting with paresthesia in the ring and little fingers, and may progress to intrinsic hand muscle weakness, atrophy, and significant functional impairment if left untreated [2].

Although conservative management may be effective in mild cases, surgical decompression is often required for patients with moderate to severe symptoms to prevent irreversible nerve damage. The two principal surgical techniques are open cubital tunnel release (OCuTR) and endoscopic cubital tunnel release (ECuTR). The open technique offers direct visualization and reliable decompression of the ulnar nerve but is associated with larger incisions, more extensive soft tissue disruption, and prolonged recovery [3-6]. In contrast, endoscopic techniques, which employ smaller incisions, aim to minimize tissue trauma and potentially reduce postoperative pain and expedite functional recovery [7].

Despite the increasing adoption of endoscopic approaches, the optimal surgical strategy remains controversial. Prior studies have reported inconsistent findings regarding comparative outcomes such as pain control, return to work, functional recovery, and complication rates [8]. Furthermore, many existing trials focus primarily on long-term endpoints, while early postoperative pain, arguably one of the most relevant metrics for evaluating minimally invasive procedures, remains underreported.

Conventional ECuTR typically involves incisions ranging from 2 to 4 cm. To further minimize soft tissue disruption, reduce the risk of medial antebrachial cutaneous (MABC) nerve injury, improve cosmetic and sensory outcomes, and accelerate early recovery, we developed a minimal incision endoscopic cubital tunnel release (MICuR) technique. In this study, "minimal incision" is defined as a subcentimeter (<1-cm) skin incision performed with standard surgical instruments and a 2.5-mm, 30-degree endoscope, without reliance on proprietary endoscopic systems. This approach aims not only to reduce surgical morbidity but also to enhance cost-effectiveness and accessibility, particularly in resource-limited settings, while preserving long-term functional outcomes [9].

This prospective randomized controlled trial compares MICuR and OCuTR in patients with cubital tunnel syndrome, focusing on short-term and mid-term outcomes including postoperative pain, time to return to work, functional recovery, scar sensitivity, and complications. By addressing key gaps in the current literature and introducing a novel technique, this study aims to refine the surgical approach to ulnar nerve decompression and enhance patient-centered outcomes.

The primary objective of this prospective randomized controlled trial was to compare short-term postoperative pain (measured by the Visual Analog Scale (VAS)) and time to return to work between MICuR and conventional OCuTR in patients with moderate to severe cubital tunnel syndrome. Secondary objectives were to evaluate functional recovery using the modified Bishop score, scar sensitivity using the Modified Vancouver Scar Scale (mVSS), and the incidence of postoperative complications up to six months after surgery.

## Materials and methods

Study design and participants

This prospective randomized controlled trial was conducted at Police General Hospital in Bangkok, Thailand, after obtaining approval from the Police General Hospital Research Ethics Board (approval number: Dh120-66) and the Thai Clinical Trials Registry (registry number: TCTR20251222016). Inclusion criteria for eligible participants were adults aged 18-60 years diagnosed with cubital tunnel syndrome classified as modified McGowan grade I or II with no prior surgical intervention for the condition. Exclusion criteria were participants with prior surgery or trauma involving the affected elbow, other peripheral nerve entrapments or generalized neurological conditions, cognitive impairment, anatomical abnormalities of the elbow (such as cubitus valgus or varus deformity greater than 10°, presence of heterotopic ossification, or significant bony deformity secondary to previous fracture that altered the normal course of the ulnar nerve), clinically evident ulnar nerve subluxation, and inability to complete postoperative follow-up evaluations. After providing written informed consent, patients were randomized into two groups: OCuTR and MICuR (Figure 1).

**Figure 1:**
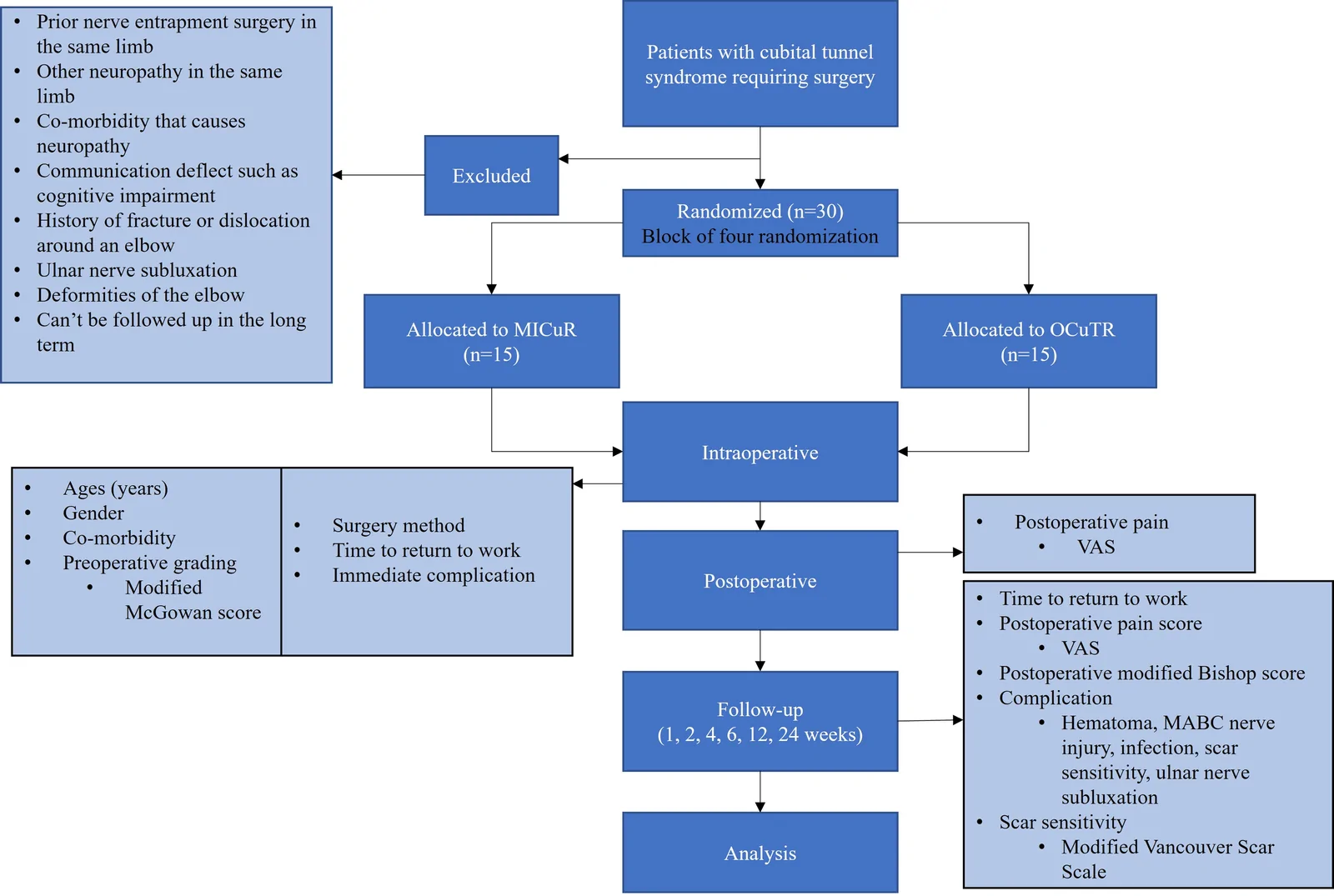
Conceptual framework MICuR: minimal incision endoscopic cubital tunnel release; OCuTR: open cubital tunnel release; VAS: Visual Analog Scale; MABC: medial antebrachial cutaneous

Diagnosis of cubital tunnel syndrome was established by a combination of clinical findings (paresthesia or numbness in the ulnar nerve distribution, positive Tinel's sign at the cubital tunnel, and/or positive elbow flexion test) and electrodiagnostic confirmation. Electrodiagnostic criteria included ulnar motor nerve conduction velocity across the elbow less than 50 m/s, and/or a significant drop in compound muscle action potential amplitude, consistent with established diagnostic standards. Severity was classified according to the modified McGowan grading system as follows: grade I: sensory symptoms only, without objective weakness or atrophy; grade IIA: sensory symptoms with mild intrinsic muscle weakness but no atrophy; and grade IIB: sensory symptoms with intrinsic muscle weakness and early atrophy. Only patients with modified McGowan grade I or II were included.

Randomization was performed using block randomization (blocks of four) via a computer-generated online program to ensure balanced allocation. All patients underwent preoperative evaluation by an anesthesiologist and received a brachial plexus block. All procedures were performed by one surgeon at Police General Hospital in Bangkok, Thailand.

With a prior retrospective cohort study comparing the outcomes of ECuTR and OCuTR [10], we determined the sample size with a 95% CI and an 80% power, increased the sample size by 10% to compensate for lost data, and then used the equation $n/group = \left( Z_{\alpha/2}\sqrt{2pq}+Z_{\beta}\sqrt{p_{1}q_{1}+p_{2}q_{2}} \right)^{2}/\left( p_{1}-p_{2} \right)^{2}$ where α=0.05, β=0.2, proportions in group 1 (treatment group)=0.764, and proportions in group 2 (control group)=0.186.

Surgical techniques

We performed OCuTR and MICuR based on the study group; both surgeries were performed by a single orthopedic hand and microsurgery specialist.

OCuTR

A 10-cm longitudinal incision was performed along the course of the cubital tunnel. The ulnar nerve and the MABC nerve were carefully identified and protected. All potential compressive structures, including the arcade of Struthers, medial intermuscular septum, medial epicondyle, Osborne ligament, and fascia of the flexor carpi ulnaris, were released. Hemostasis was secured, and the wound was closed with subcuticular 4-0 monofilament sutures and reinforced with Steri-Strips (Figure 2).

**Figure 2:**
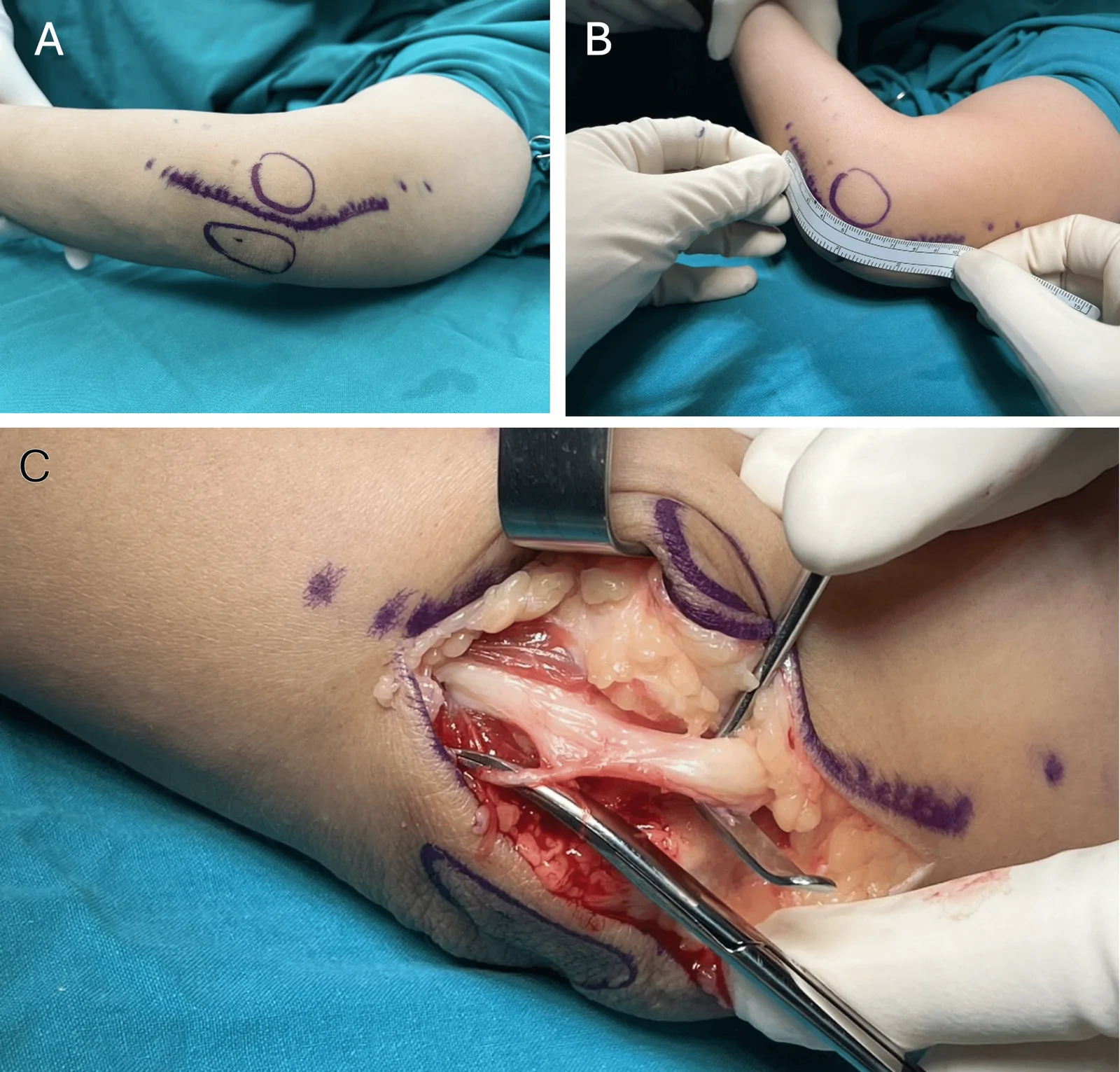
Standard open cubital tunnel release (A) Surgical landmarks of open cubital tunnel release, with the incision made between the medial humeral condyle and olecranon. (B) The incision length of open cubital tunnel release, measured about 10 centimeters. (C) Ulnar nerve underlying the cubital tunnel and its branch.

MICuR

We started by drawing surgical landmarks determined by the medial epicondyle and olecranon, and then a 1-cm skin incision was placed directly over the cubital tunnel between the medial epicondyle and olecranon. Blunt dissection was performed using Metzenbaum scissors, and the soft tissues were retracted with a Senn retractor before a 2.5-mm, 30-degree endoscope was introduced, enabling the direct visualization of the ulnar nerve and surrounding structures. After the introduction of the 2.5-mm, 30-degree endoscope, the soft tissue envelope was gently elevated with a Senn retractor. Curved Metzenbaum scissors were advanced under continuous endoscopic visualization. The extent of release (approximately 7 cm proximal and 7 cm distal to the medial epicondyle) was estimated using the known length markings on the scissors and confirmed by direct endoscopic visualization of the released fascia and the freely mobile ulnar nerve. The proximal limit reached the arcade of Struthers region and the distal limit extended beyond the flexor carpi ulnaris aponeurosis. After adequate decompression was achieved, the incision was closed with 4-0 monofilament sutures and reinforced with Steri-Strips. In the event of intraoperative ulnar nerve subluxation, the operation was switched to open ulnar nerve transposition [9] (Figures 3-4).

**Figure 3:**
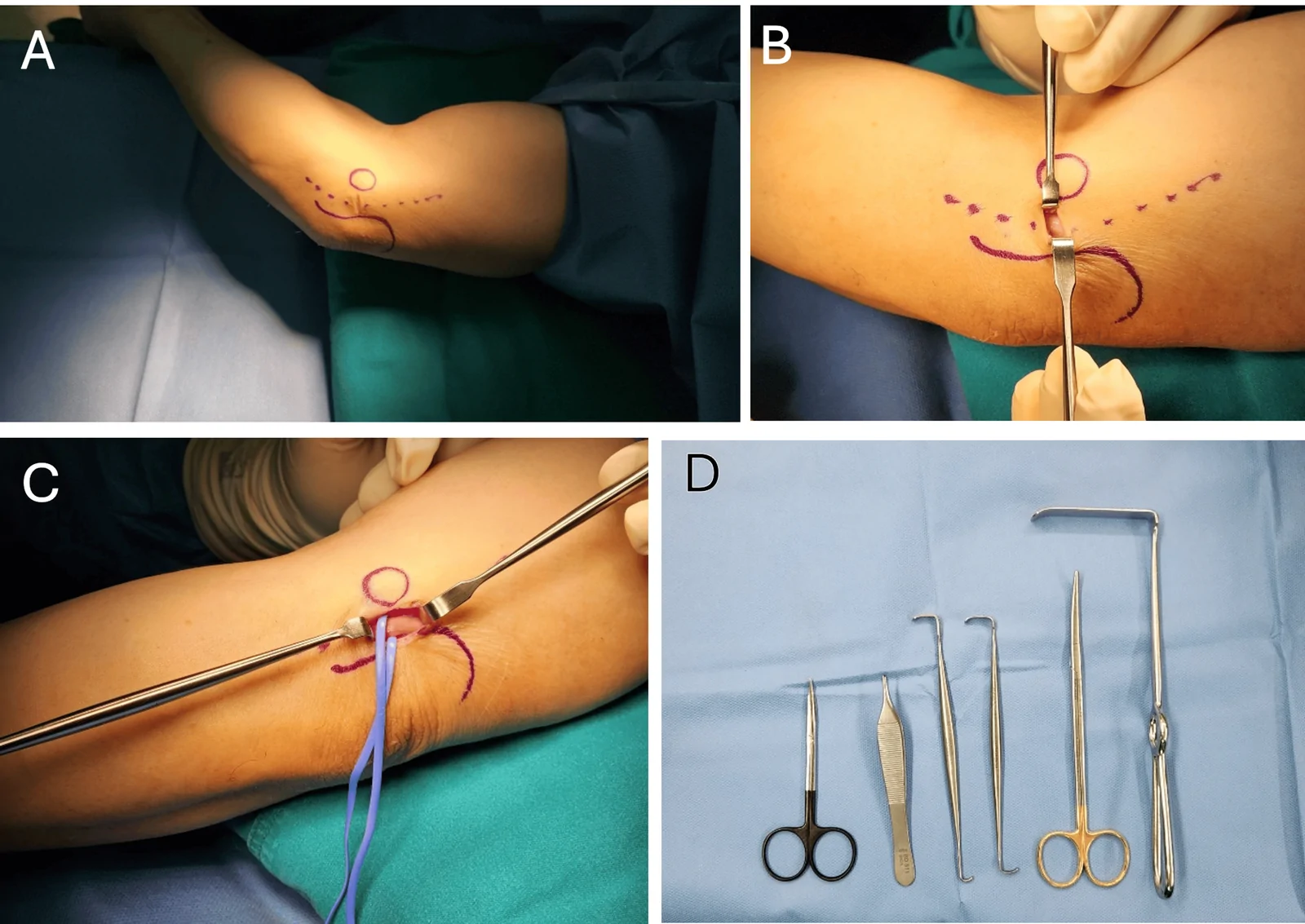
Minimal incision endoscopic cubital tunnel release (A) Surface anatomy landmarks of the medial humeral condyle, ulnar nerve, and olecranon. (B) The incision in minimal incision endoscopic cubital tunnel release, measured about 1 centimeter. (C) Ulnar nerve underlying the cubital tunnel, identified via minimal incision. (D) Surgical instruments in minimal incision endoscopic cubital tunnel release (from left to right: Metzenbaum scissors, forceps, Senn retractor, curved short-blade Metzenbaum scissors, and Langenbeck retractor).

**Figure 4:**
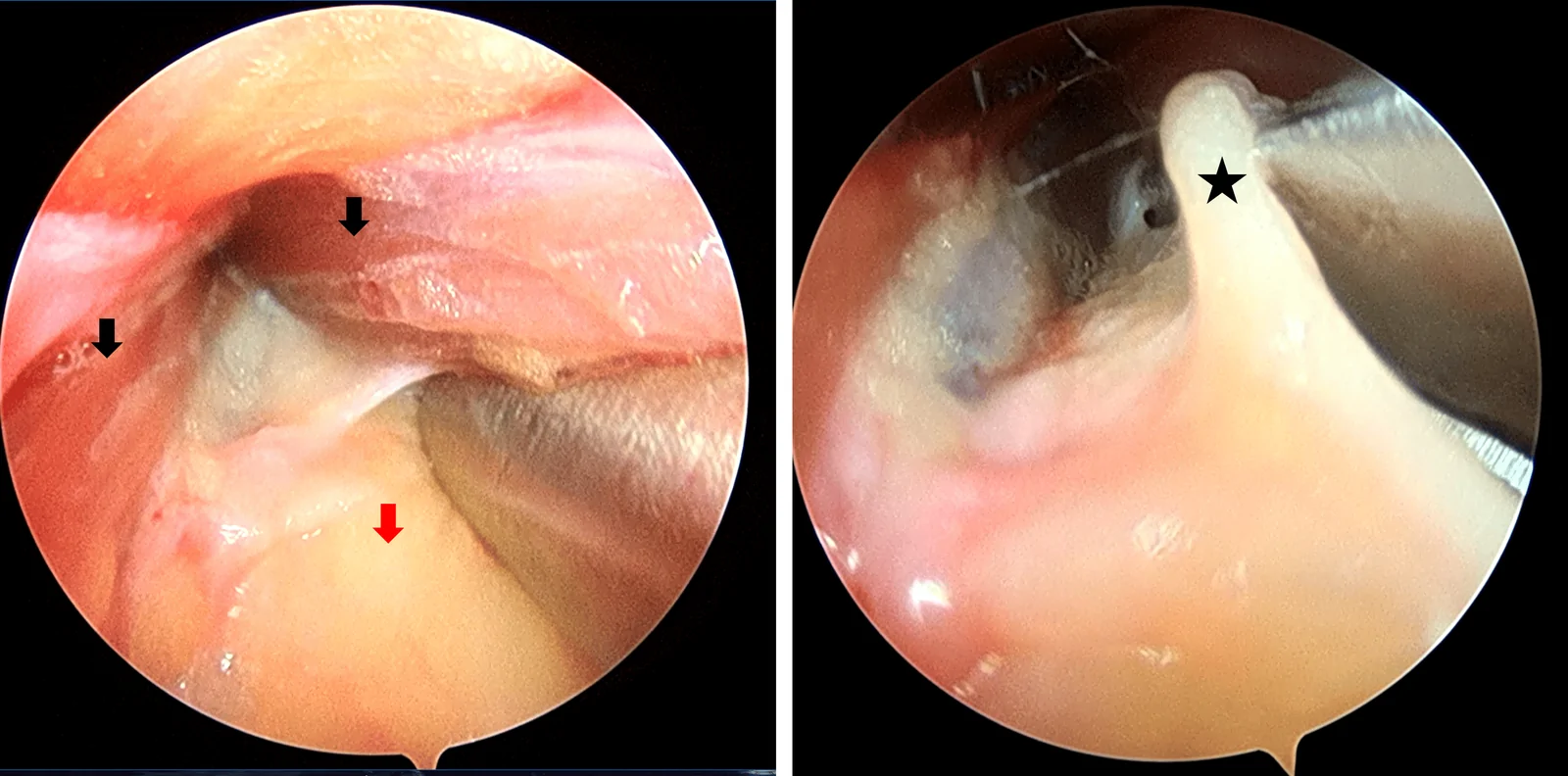
Endoscopic view of the minimal incision endoscopic cubital tunnel release technique Direct visualization of the flexor carpi ulnaris (black arrows) and the ulnar nerve (red arrow) with minimal incision under endoscope. The medial antebrachial cutaneous nerve (star) can be visualized under endoscope, and injury to the medial antebrachial cutaneous nerve can be avoided.

All procedures were performed under ultrasound-guided brachial plexus block by an experienced anesthesiologist. For the MICuR technique, a standard 2.5-mm, 30-degree rigid endoscope was used together with conventional Metzenbaum scissors, a Senn retractor, and a Langenbeck retractor. No proprietary endoscopic cubital tunnel system was employed. Both operations were done under tourniquet.

Postoperative protocol and outcome measures

Pain management, wound care, and rehabilitation were standardized for both groups. Analgesia consisted of oral paracetamol 500 mg every six hours as needed and a non-steroidal anti-inflammatory drug (ibuprofen 400 mg every eight hours) for the first five days; stronger opioids were not routinely prescribed. Wound care involved a sterile dressing changed on postoperative day 2, followed by daily inspection and application of adhesive strips until suture removal at 14 days. Rehabilitation began on the day of surgery with active finger and wrist motion. Patients were instructed to avoid prolonged elbow flexion beyond 90° for the first two weeks and were encouraged to resume daily activities as tolerated. Formal physiotherapy was not routinely prescribed. Clinical evaluations were conducted at the following postoperative intervals: 0-7 days, two weeks, one month, three months, and six months.

All postoperative assessments were performed by an independent orthopedic resident of Police General Hospital in Bangkok, Thailand, who was fully blinded to the surgical approach and performed all postoperative assessments. Patients were also instructed not to reveal their treatment modality, ensuring the integrity of assessor blinding. The primary outcomes included time to return to work and postoperative pain assessed using the VAS (Table 1). Furthermore, we recorded functional outcomes measured by the modified Bishop score (Table 2) [11], scar sensitivity evaluated using the mVSS (Table 3) [12], and postoperative complications as secondary outcomes.

**Table 1:** Visual Analog Scale

	Day(s)							2 weeks	1 month	3 months	6 months
	1	2	3	4	5	6	7				
Visual Analog Scale											

**Table 2:** Modified Bishop score

Modified Bishop score	Score
Residual symptoms	
None	3
Little/intermitted	2
Moderate	1
Severe	0
Subjective improvement	
Better	2
Unchanged	1
Worse	0
Ability to work	
Resumed previous job	2
Changed job due to complaints	1
Incapable of working	0
Muscle strength	
Better	1
Unchanged	0
Evaluation	
Excellent	8-9
Good	6-7
Fair	4-5
Poor	<3

**Table 3:** Modified Vancouver Scar Scale

Scar characteristics	Score
Vascularity	
Normal	0
Pink	1
Red	2
Purple	3
Pigmentation	
Normal	0
Hypopigmentation	1
Mixed	2
Hyperpigmentation	3
Pliability	
Normal	0
Supple	1
Yielding	2
Firm	3
Ropes	4
Contracture	5
Height	
Flat	0
<2 mm	1
2-5 mm	2
>5 mm	3
Pain	
None	0
Occasional	1
Requiring medication	2
Pruritus	
None	0
Occasional	1
Requiring medication	2
Total score	16

Statistical analysis was performed using IBM SPSS Statistics for Windows, Version 29.0 (IBM Corp., Armonk, New York, United States). Continuous variables were compared with the independent Student's t-test (or Mann-Whitney U test if non-normally distributed) and categorical variables with the chi-squared or Fisher's exact test as appropriate. A two-sided p-value of <0.05 was considered statistically significant. Because the primary outcomes (postoperative pain and time to return to work) were pre-specified, no formal multiplicity adjustment was applied. All analyses were conducted on an intention-to-treat basis. There were no missing data or dropouts during the six-month follow-up period. 

## Results

Demographic data

A total of 35 participants were enrolled in the study from March 2024 to February 2025. Five were excluded for the following reasons: history of prior surgery, history of fracture, can't comply with follow-up, and co-morbidity interfering with neuropathy. The rest were randomized equally into two groups: 15 patients underwent MICuR and 15 underwent OCuTR. The mean age was 51 years in the MICuR group and 48 years in the OCuTR group (p=0.6717 at 95% CI). The majority of participants in both groups were classified as modified McGowan grade II. Baseline characteristics, including co-morbidities such as diabetes mellitus and hypertension, were comparable between the two groups with no statistically significant differences (Table 4). All 30 participants were able to follow up until the last visit; there was no dropout in this study.

**Table 4:** Statistic comparison of demographic data in the OCuTR and MICuR groups Continuous data, such as age, were analyzed using the independent t-test. Other descriptive data were analyzed using the chi-squared test. MICuR: minimal incision endoscopic cubital tunnel release; OCuTR: open cubital tunnel release

Demographic data	OCuTR	MICuR	P-value (95% CI)
Sex			
Male	7	5	0.456
Female	8	10	
Age (years)	48	51	0.6717
Side			
Right	8	9	0.713
Left	7	6	
Grade			
I	1	3	0.283
IIa	7	8	0.715
IIb	7	4	0.256
Underlying disease			
Diabetes mellitus	2	1	0.543
Hypertension	1	2	0.543
Dyslipidemia	2	5	0.195
Spinal disease	2	0	0.143
Rheumatoid arthritis	0	1	0.309
Ischemic heart disease	0	1	0.309

Postoperative outcomes

Patients in both groups were advised to return to work with their normal activities after surgery when comfortable. The MICuR group returned to work significantly earlier than those in the OCuTR group. The mean time to return was two days for the MICuR group and 13 days for the OCuTR group (p<0.01 at 95% CI), indicating a substantial advantage in early recovery for the endoscopic technique (Table 5).

**Table 5:** Statistic comparison of the average time to return to work (day) and number of complications that occurred in the OCuTR and MICuR groups Time to return to work was analyzed using Student's t-test. Incidences of complications were analyzed using the chi-squared test. MICuR: minimal incision endoscopic cubital tunnel release; OCuTR: open cubital tunnel release; MABC: medial antebrachial cutaneous

	OCuTR	MICuR	P-value (95% CI)
Time to return to work (day)	13	2	<0.01
Complication			
MABC nerve injury (case)	3	0	0.068
Hematoma (case)	1	2	0.543
Ulnar nerve subluxation (case)	2	1	0.543
Infection (case)	0	0	1

Postoperative pain was significantly lower in the MICuR group through day 1 up to the one-month follow-up, and the result became comparable after the three-month follow-up (Table 6 and Figure 5). These findings highlight the short-term analgesic benefit of the MICuR technique during the early postoperative period. Furthermore, the scar sensitivity was also significantly lower in the MICuR group (3.2 vs. 9.7, 1.9 vs. 6.2, and 0.0 vs. 2.5 at one, three, and six months, respectively; p<0.01 at 95% CI for all comparisons) (Table 6 and Figure 6). Both groups demonstrated progressive functional improvement throughout the six-month follow-up. Although the MICuR group consistently showed slightly higher scores, the differences were not statistically significant at any timepoint (Table 6 and Figure 7).

**Table 6:** Statistic comparison of postoperative pain via VAS, modified Bishop score, and scar sensitivity via mVSS in the OCuTR and MICuR groups Postoperative pain, modified Bishop score, and scar sensitivity were analyzed using Student's t-test. MICuR: minimal incision endoscopic cubital tunnel release; OCuTR: open cubital tunnel release; VAS: Visual Analog Scale; mVSS: Modified Vancouver Scar Scale

Time	Postoperative pain (VAS)			Modified Bishop score			Scar sensitivity (mVSS)		
	OCuTR	MICuR	P-value (95% CI)	OCuTR	MICuR	P-value (95% CI)	OCuTR	MICuR	P-value (95% CI)
1 day	4.73	0.8	<0.01	-	-	-	-	-	-
2 days	4.47	0.47	<0.01	-	-	-	-	-	-
3 days	3.47	0.2	<0.01	-	-	-	-	-	-
4 days	3.2	0	<0.01	-	-	-	-	-	-
5 days	2.2	0	<0.01	-	-	-	-	-	-
6 days	1.87	0	<0.01	-	-	-	-	-	-
1 week	1.73	0	<0.01	5.53	7.67	1	-	-	-
2 weeks	1	0	0.02	5.67	7.93	1	-	-	-
1 month	0.47	0	0.03	6.47	8.2	1	9.73	3.2	<0.01
3 months	0.2	0	0.07	7.07	8.53	0.99	6.27	1.93	<0.01
6 months	0.2	0	0.07	7.6	8.53	0.99	2.47	0	<0.01

**Figure 5:**
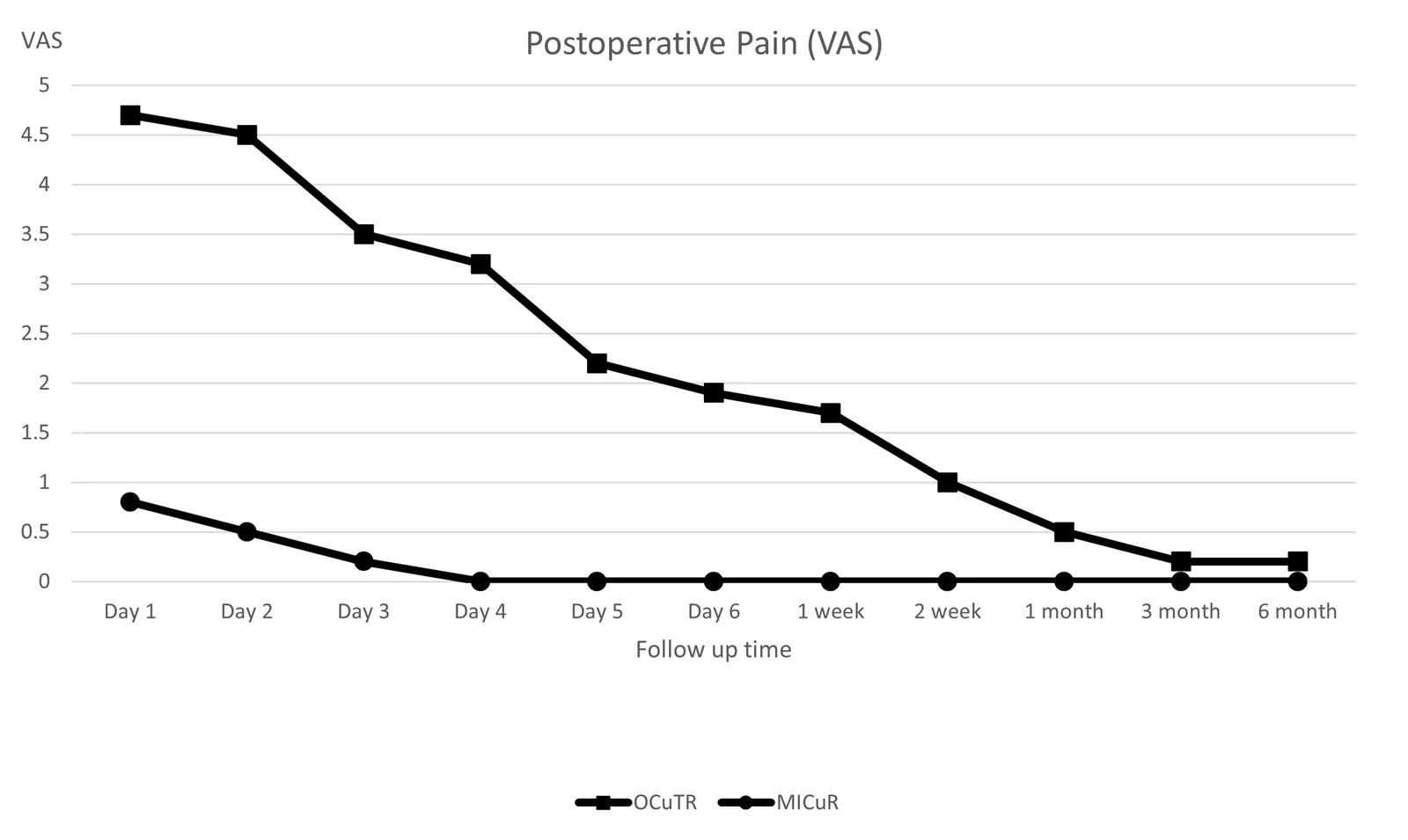
Comparison of postoperative pain via VAS in the OCuTR and MICuR groups at one day to six months of follow-up Postoperative pain was analyzed using Student's t-test. MICuR: minimal incision endoscopic cubital tunnel release; OCuTR: open cubital tunnel release; VAS: Visual Analog Scale

**Figure 6:**
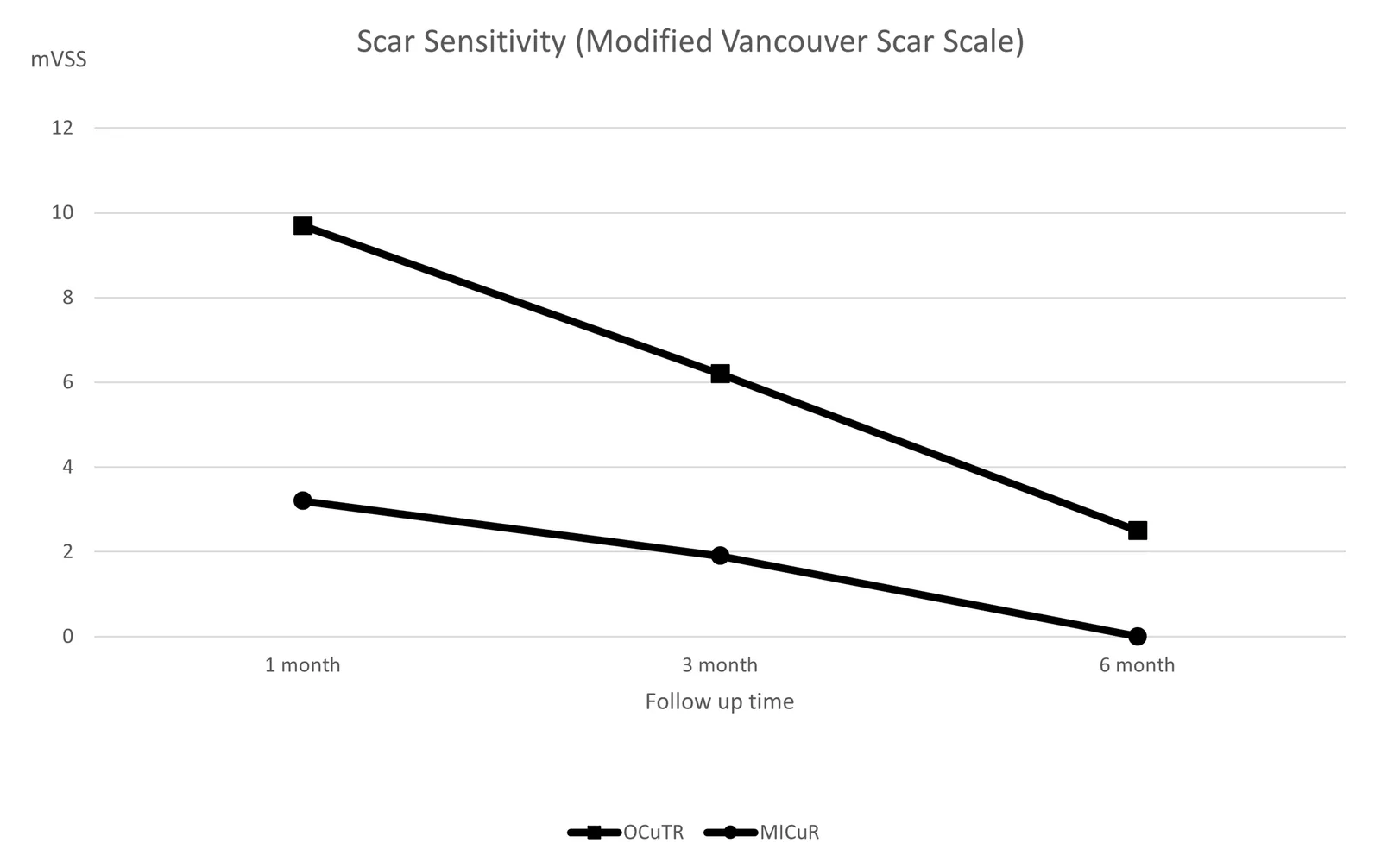
Comparison of scar sensitivity via mVSS in the OCuTR and MICuR groups at one to six months of follow-up Scar sensitivity by mVSS was analyzed using Student's t-test. MICuR: minimal incision endoscopic cubital tunnel release; OCuTR: open cubital tunnel release; mVSS: Modified Vancouver Scar Scale

**Figure 7:**
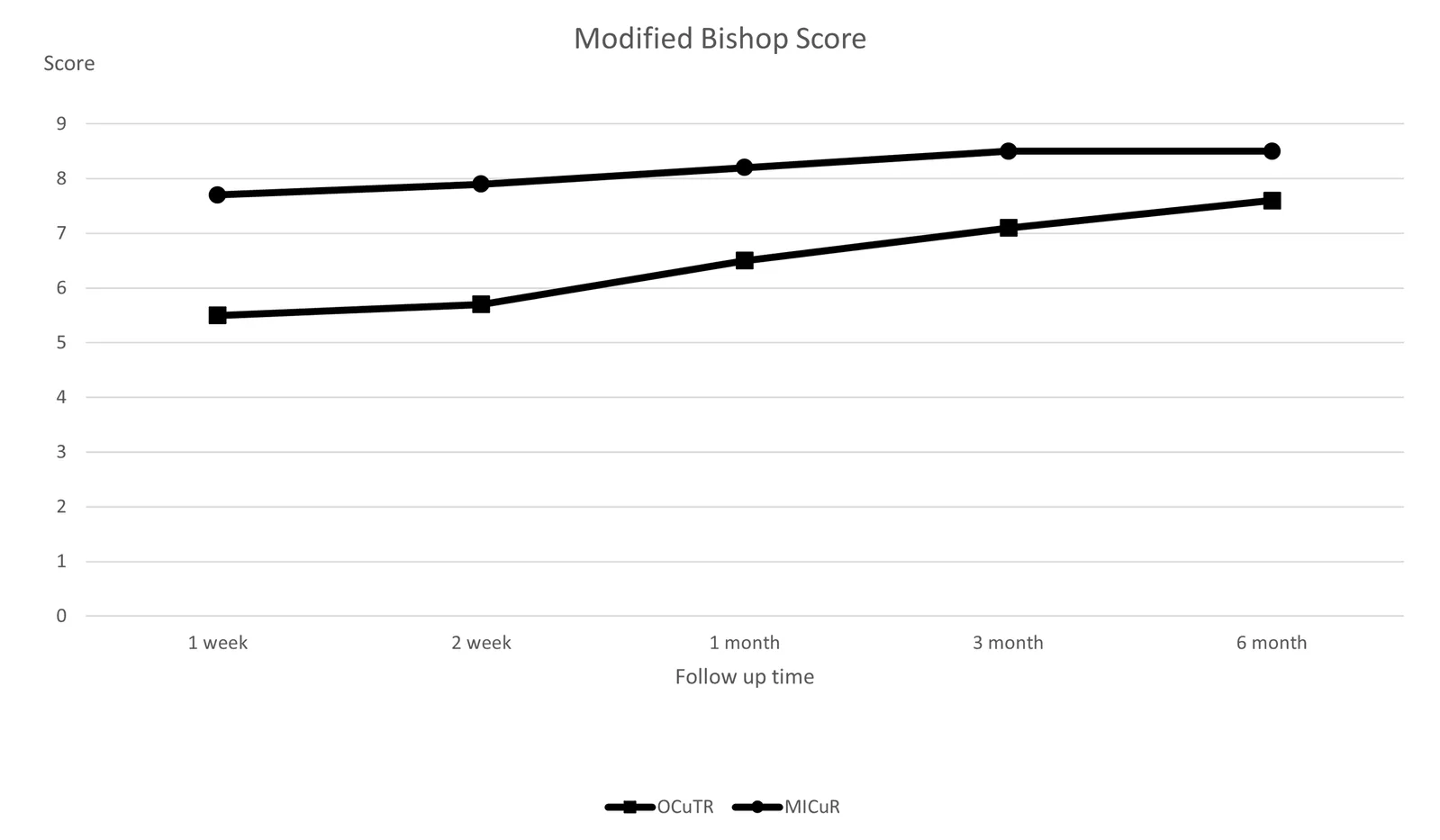
Comparison of modified Bishop score in the OCuTR and MICuR groups at one week to six months of follow-up Modified Bishop score was analyzed using Student's t-test. MICuR: minimal incision endoscopic cubital tunnel release; OCuTR: open cubital tunnel release

As for complications, MABC nerve injury was observed in three patients (20%) in the OCuTR group and none in the MICuR group (p=0.068 at 95% CI), hematoma occurred in two MICuR cases and one OCuTR case (p=0.543 at 95% CI), ulnar nerve subluxation was reported in two patients in the OCuTR group and one in the MICuR group, and no infection was reported in either group (Table 5). While none of the differences reached statistical significance, the absence of MABC nerve injury in the MICuR group suggests a trend toward reduced iatrogenic nerve trauma.

## Discussion

Minimally invasive approaches for cubital tunnel syndrome have gained considerable interest in recent years due to their potential to reduce soft tissue disruption, alleviate postoperative discomfort, and promote faster recovery. However, despite these theoretical advantages, previous comparative studies of endoscopic versus open decompression techniques have produced inconsistent results particularly regarding functional outcomes, complication rates, and pain reduction [4,6,10,13]. A major limitation of the existing literature is the limited reporting of early postoperative pain, as well as the relatively large incisions (2-4 cm) used in conventional endoscopic procedures, which may diminish the purported advantages of minimally invasive surgery.

Our study sought to address these gaps by implementing a standardized outcome assessment beginning on postoperative day 1, extending through a six-month follow-up period. We compared the outcomes of a novel MICuR technique, using a sub-1-cm incision and standard instruments, against the conventional OCuTR. The findings suggest that MICuR offers meaningful short-term benefits over the traditional open approach, without compromising safety or functional recovery.

Among the most notable findings was the significantly shorter time to return to work in the MICuR group (two days vs. 13 days; p<0.01 at 95% CI), supporting the notion that reduced surgical trauma facilitates earlier resumption of daily activities. This aligns with earlier reports demonstrating accelerated recovery following endoscopic techniques [6,14,15] and has important implications for working-age populations, healthcare utilization, and overall cost-effectiveness [9].

Early postoperative pain was also markedly lower in the MICuR group. On postoperative day 1, VAS scores averaged 0.8 compared to 4.7 in the OCuTR group (p<0.01 at 95% CI). This analgesic advantage persisted for several weeks before diminishing, with no statistically significant difference at six months. These results emphasize the importance of early pain assessment in evaluating minimally invasive techniques, an aspect often underreported in prior trials. Meta-analyses and systematic reviews may have underestimated this transient yet clinically meaningful benefit by focusing solely on mid- to long-term outcomes [4,5,13].

Another key finding was the significantly lower scar sensitivity reported in the MICuR group, as measured by mVSS. Scores were consistently lower at one, three, and six months, with MICuR patients approaching zero by the final follow-up. While often overlooked in surgical outcomes research, cosmetic and sensory aspects of surgical scars can substantially impact patient satisfaction, particularly in younger and more active individuals [15,16].

Functional recovery, as assessed by the modified Bishop score, improved gradually and comparably in both groups. This reinforces prior findings suggesting that long-term function does not differ significantly between open and endoscopic techniques [6,16-18].

Complication rates were low and similar between groups, although certain trends were observed. While the difference in MABC nerve injury rates did not reach statistical significance (p=0.068), the 20% incidence observed in the OCuTR group is clinically relevant. Injury to the MABC nerve can cause persistent numbness, dysesthesia, or neuropathic pain over the medial elbow and proximal forearm, which may reduce patient satisfaction. The complete absence of MABC nerve injury in the MICuR group suggests a potential advantage, possibly reflecting increased soft tissue disruption and longer exposure in the open procedure [19,20], although larger studies are needed to confirm this observation. Conversely, hematomas occurred slightly more often in the MICuR group (two cases vs. one), a finding consistent with previous endoscopic studies, where limited visualization may increase the risk of minor bleeding [21,22]. Importantly, no infections or reoperations were reported in either group.

A key innovation of the MICuR technique is the use of a sub-1-cm incision without reliance on proprietary endoscopic systems. While many existing endoscopic methods utilize specialized tools such as the Agee system or dedicated retractors [21,23,24], our method employs standard surgical instruments and a 2.5-mm, 30-degree endoscope. This approach may enhance its scalability and adoption in resource-limited settings [9].

Additionally, the procedure was performed safely and effectively under brachial plexus block anesthesia, with no intraoperative complications or conversions to open surgery. This demonstrates the feasibility of MICuR in routine clinical practice, provided surgeons possess adequate training in endoscopic anatomy and nerve decompression techniques [20,23,25].

While the early recovery and cosmetic advantages of MICuR are compelling, long-term outcomes remain a critical consideration. Our six-month follow-up does not capture the potential for recurrence, delayed complications, or late-stage nerve regeneration. Future multicenter trials with longer follow-up and incorporation of objective measures such as nerve conduction studies and grip strength will be essential to validate the durability and generalizability of MICuR [13,18].

Study limitations

While MICuR demonstrated clear short-term benefits, several limitations must be acknowledged. First, the modest sample size (n=30) limits the statistical power and generalizability of the findings. Second, long-term functional outcomes such as grip strength, nerve conduction studies, and recurrence rates were not assessed, which restricts conclusions regarding the durability of the procedure. Third, cost-effectiveness was not formally evaluated; however, the use of standard surgical instruments in MICuR may enhance its scalability and accessibility compared to proprietary endoscopic systems. 

All procedures were performed by a single experienced hand surgeon at a single tertiary center. This design minimizes technical variability but introduces potential selection bias and limits the generalizability of the findings to community practice or to surgeons with less experience in endoscopic nerve decompression. Results may differ when the MICuR technique is performed by surgeons still on the learning curve or in different institutional settings with varying patient populations and resources.

## Conclusions

This prospective randomized controlled trial suggests that MICuR provides meaningful short-term advantages over conventional OCuTR, including significantly faster return to work, reduced early postoperative pain, and lower scar sensitivity without an observed increase in complications. Functional outcomes at six months were comparable between groups.

These results support MICuR as a viable and potentially preferable surgical option for patients prioritizing rapid recovery and cosmetic outcomes. However, due to the study's limited sample size, single-surgeon design, and short follow-up duration, further multicenter trials with larger cohorts and long-term evaluation are warranted to validate these findings and clarify the broader role of MICuR in the surgical treatment of cubital tunnel syndrome.
